# Preoperative multimodal prehabilitation before elective colorectal cancer surgery in patients with WHO performance status I or II: randomized clinical trial

**DOI:** 10.1093/bjsopen/zrad134

**Published:** 2023-12-07

**Authors:** Rasmus Dahlin Bojesen, Susanne Oksbjerg Dalton, Søren Thorgaard Skou, Lars Bo Jørgensen, Line Rosell Walker, Jens Ravn Eriksen, Camilla Grube, Tobias Freyberg Justesen, Christoffer Johansen, Gerrit Slooter, Franco Carli, Ismail Gögenur

**Affiliations:** Department of Surgery, Slagelse Hospital, Slagelse, Denmark; Center for Surgical Science, Zealand University Hospital, Køge, Denmark; Department of Clinical Oncology, Zealand University Hospital, Næstved, Denmark; The Danish Cancer Society Research Center, Copenhagen, Denmark; Research Unit for Musculoskeletal Function and Physiotherapy, Department of Sports Science and Clinical Biomechanics, University of Southern Denmark, Odense, Denmark; The Research Unit PROgrez, Department of Physiotherapy and Occupational Therapy, Naestved-Slagelse-Ringsted Hospitals, Slagelse, Denmark; Research Unit for Musculoskeletal Function and Physiotherapy, Department of Sports Science and Clinical Biomechanics, University of Southern Denmark, Odense, Denmark; The Research Unit PROgrez, Department of Physiotherapy and Occupational Therapy, Naestved-Slagelse-Ringsted Hospitals, Slagelse, Denmark; Department of Physiotherapy and Occupational Therapy, Zealand University Hospital, Roskilde, Denmark; Department of Surgery, Slagelse Hospital, Slagelse, Denmark; Center for Surgical Science, Zealand University Hospital, Køge, Denmark; Department of Surgery, Zealand University Hospital, Køge, Denmark; Department of Surgery, Slagelse Hospital, Slagelse, Denmark; Center for Surgical Science, Zealand University Hospital, Køge, Denmark; Center for Surgical Science, Zealand University Hospital, Køge, Denmark; The Danish Cancer Society Research Center, Copenhagen, Denmark; Late Effect Research Unit CASTLE, Finsen Center, Rigshospitalet, Copenhagen, Denmark; Department of Surgery, Maxima Medical Center, Eindhoven, The Netherlands; Department of Anesthesia, Faculty of Medicine and Health Sciences, McGill University, Montreal, Quebec, Canada; Center for Surgical Science, Zealand University Hospital, Køge, Denmark

## Abstract

**Background:**

Multimodal prehabilitation is a promising adjunct to the current surgical treatment pathway for colorectal cancer patients to further improve postoperative outcomes, especially for high-risk patients with low functional capacity. The aim of the present study was to test the effect of prehabilitation on immediate postoperative recovery.

**Method:**

The study was designed as a RCT with two arms (intervention and control). The intervention consisted of 4 weeks of multimodal prehabilitation, with supervised physical training, nutritional support and medical optimization. The control group received standard of care. A total of 40 patients with colorectal cancer (WHO performance status I or II) undergoing elective surgery with curative intent were included. The primary outcome was postoperative recovery within the first 3 postoperative days, measured by Quality of Recovery-15, a validated questionnaire with a scoring range between 0 and 150 and a minimal clinically relevant difference of 8.

**Results:**

In total, 36 patients were analysed with 16 in the intervention group and 20 in the control group. The mean age of the included patients was 79 years. The overall treatment effect associated with the intervention was a 21.9 (95% c.i. 4.5–39.3) higher quality of recovery-15 score during the first 3 postoperative days compared to control, well above the minimal clinically relevant difference.

**Conclusion:**

Four weeks of multimodal prehabilitation prior to elective curative intended colorectal cancer surgery in patients with WHO performance status I or II was associated with a clinically relevant improvement in postoperative recovery.

Registration number: NCT04167436 (http://www.clinicaltrials.gov)

## Teaser

In this randomized clinical trial comparing patients undergoing 4 weeks of multimodal prehabilitation prior to elective colorectal cancer surgery with usual care, prehabilitation was associated with a clinically relevant improvement in postoperative recovery. The overall treatment effect associated with the intervention was an improvement in the quality of recovery-15 score of 21.9 (95% c.i. 4.5–39.3) during the first 3 postoperative days compared to control.

## Introduction

Multimodal prehabilitation through exercise, nutritional counselling and medical optimization prior to surgery is a promising addition to the existing enhanced recovery after surgery (ERAS)^1^ pathway to further improve surgical outcomes^2^. While definitive results of the ability of multimodal prehabilitation to reduce postoperative complications are currently lacking^3,4^, the recent published PREHAB trial showed a reduction in postoperative medical complications for patients undergoing multimodal prehabilitation prior to elective colorectal cancer surgery^5^. Several large-scale studies are currently being conducted with a reduction in postoperative complications as their primary outcome. These studies build on the theoretical framework for prehabilitation initially proposed by Carli and Zavorsky^6^ in the early 2000s. In this model, improvements in functional capacity prior to surgery will lead to less time spent below the threshold of dependency, improving postoperative recovery and reducing the risk of postoperative complications. While several studies have shown that it is possible to improve patients’ functional capacity through prehabilitation^5,7^, no previous studies have tested if this increase will actually lead to improved early recovery. This assumption is of major importance for both understanding the effects of prehabilitation and confirming causal inference between improving functional outcomes and reduction in complications.

Functional capacity is the ability to perform daily activities and self-care, and patients with low functional capacity have increased risk of both morbidity and mortality after surgery^8–10^. In this model, an intrinsic assumption is that patients with lower functional capacity are closer to the threshold of dependency and thereby potentially have most to gain from preoperative interventions^6^. The WHO performance status is a commonly used tool for assessing functional capacity in oncology that is associated with increased risk of both complications and short- and long-term mortality after colorectal cancer surgery^11^.

The aim of this study was to estimate the effect of multimodal prehabilitation intervention on postoperative recovery in patients with WHO performance status I or II, prior to elective surgery for colorectal cancer.

## Methods

### Study design and setting

The study was conducted as a RCT between an intervention group and a control group of 40 patients in a 1:1 ratio. Patients were recruited from two centres that cover the entire health region of Region Zealand, Denmark: the Department of Surgery, Zealand University Hospital and the Department of Surgery, Slagelse Hospital. The study period was from 25 April 2019 to 5 May 2022. The study was conducted as an individual substudy within the international multicentre study PREHAB^5^ (NTR5947), including only Danish centres, more restrictive eligibility criteria and with a different primary outcome, although using the same interventions as the primary trial.

### Study population

The study population consisted of patients planned for elective surgery with curative intent for colorectal cancer with low functional capacity. This was assessed by the treating surgon and patients with WHO performance status I (ambulatory, but restricted in physically strenuous activities) or II (ambulatory, but unable to carry out any work activities, sedentary less than 50% of waking hours)^11^ were included. Exclusion criteria were inability to understand Danish, chronic renal failure (creatinine ≥250 µmol/l), inability to perform baseline tests or inability to perform training on a stationary exercise bike, severe cognitive impairment (Mini-Mental State Examination ≤11^12^), planned for abdominal perineal resection, subacute surgery, neoadjuvant therapy, metachronous cancer, or withdrawal of consent.

### Interventions

The study was approved by the Scientific Ethical Committee (Jr.: SJ-607) and all patients signed informed consent forms before inclusion. The full intervention has been published in detail elsewhere to ensure replication^13^, as this study was preceded by a feasibility trial. In short, the interventions consisted of individualized physical training three times a week (Monday, Wednesday and Friday) for a minimum of 4 weeks, a nutritional intervention consisting of both nutritional supplements and consultation with a dietician, and medical optimization through an expanded medical check-up at inclusion. The training intervention consisted of supervised high-intensity interval training (HIIT) on a stationary exercise bike. Patients were instructed to perform four high-intensity bouts of 2–3 min at a wattage achieved at 90% of the patient’s maximum oxygen uptake measured at baseline with a cardiopulmonary exercise test. Between each high-intensity bout, 3 min of low-intensity bouts (30% of the maximum oxygen uptake) were performed. The HIIT was followed by resistance training of the large muscle groups of three sets of 8–12 repetitions, following a predefined progression scheme, using machines (Technogym®, Italy).

The nutritional intervention consisted of a consultation with a dietician within the first week after inclusion of approximately 1 h in length and a uniform prescription of protein supplements (30 g of protein twice a day using the TMP-90 Shake® (Friesland Campina, Netherlands)) and vitamins (vitamin D with calcium (38 µg + 400 mg Unikalk Mega®, Orkla Health A/S, Denmark) and multivitamin (Apovit Multi®, Apovit, Denmark)).

The expanded medical check-up was conducted at baseline by the principal investigator and included evaluation of baseline tests, bloodwork, referral to alcohol and smoking cessation, and possible drug discontinuation or dose reduction using the Danish health authority’s guidelines^14^. If any suspicion of an unknown or poorly regulated disease was present, the patient was referred to a specialist.

The control group received standard of care defined by local and national guidelines for the surgical treatment of colorectal cancer at both centres. This included intravenous iron for anaemic patients (haemoglobin <11.28 g/dl (7 mmol/l) for both men and women) with a postponement of surgery approximately 4 weeks after initial infusion, preoperative lung physiotherapy, patient education, limited fasting, carbohydrate loading and full enhanced recovery after surgery as described by the 2018 Guidelines for colorectal cancer^1^. Discharge was handled by standardized discharge criteria for both groups.

### Randomization and blinding

Randomization was conducted by the *researchmanager* software (Research manager, Netherlands) in random blocks of eight. No means of stratification of patients was used. Patients, investigators and assessors were not blinded, as this was not feasible. However, randomization was conducted immediately after baseline testing leading to the blinding of both the participant and the investigators at the baseline test.

### Testing

Patients were tested at inclusion (baseline), just prior to surgery (preoperative) and 4 weeks after surgery (postoperative). All testing sessions consisted of questionnaires (European Organisation for Research and Treatment of Cancer Quality of Life-29/Core30^15^, Short Form-36^16^, Geriatric 8^17^, Patient Health Questionaire-9^18^, General Anxiety Disorder-7^19^ and Patient-Generated Subjective Global Assessment^20^), anthropometric measurements, blood work, a cardiopulmonary exercise test^21^, hand grip strength, isometric leg extension strength test^22^, 6 minutes’ walk test (6-MWT)^23^, 30 s sit to stand test^24^ and 30 s stair climb test (number of steps taken on a stairwell in 30 s), in this exact order at each visit.

### Primary outcome

The primary outcome of the study was postoperative recovery during the first 3 postoperative days measured by Quality of Recovery 15 questions (QoR-15)^25^. QoR-15 consists of 15 questions aimed at common postoperative conditions such as pain, nausea/vomiting, exhaustion, anxiety and overall well-being, and estimates the time spent with each condition during the last 24 h on a scale from 0 to 10, with a maximum total score of 150. QoR-15 has been translated and validated in the Danish surgical population^26^, with a minimal clinically relevant difference of 8^27^, and can be used as a continuous variable. The severity of the QoR-15 score is associated with both postoperative complications and return to recreational and occupational activities^28^.

### Secondary outcomes

The study had several predefined secondary outcomes including changes in physical fitness, physical function, nutritional status, blood work between baseline and preoperative testing sessions, and postoperative complications. The changes in physical fitness were measured as VO_2_ max (ml/kg/min) and VO_2_ (ml/kg/min) at the anaerobic threshold through the cardiopulmonary exercise test. Changes in physical function were measured through the 6-MWT, 30 s sit to stand test and the hand grip strength test. Nutritional status was evaluated through changes in total body weight and the patient-generated subjective global assessment (PG-SGA) score. Changes in bloodwork were estimated in regards to haemoglobin (mmol/l), neutrophil-to-lymphocyte ratio, C-reactive protein (mg/l), creatinine (µmol/l) and albumin (g/l). Postoperative complications were evaluated 30 days after surgery and graded both by the Clavien–Dindo classification^29^ and the Comprehensive Complication Index^30^, by a blinded external assessor.

### Other variables

Adherence to the training intervention was measured as the number of attended training sessions out of the maximum possible, and compliance was measured as the number of training sessions meeting a minimum of 4 min above the 90% threshold for HIIT. Compliance with the nutritional intervention was measured as the percentage of the ingested protein at the preoperative testing. No predefined measure of compliance or adherence to the medical optimization was conducted. Adverse events during the training intervention were defined as any condition arising during the preoperative phase needing medical attention or treatment. Expected events such as muscle soreness were not registered. Compliance with ERAS was measured by urinary catheter removal, initiation of normal diet and postoperative day of full mobilization defined by achieving baseline mobility. Length of stay was estimated from day of surgery until day of discharge, and by days alive and out of hospital (DAOH-30)^31^. A readmission was defined as any unplanned admission to any hospital within 30 days after surgery of more than 24 h in length.

### Sample size and impact of the coronavirus disease 2019 pandemic

Before the initiation of the study, a sample size calculation was conducted using the primary outcome QoR-15, with a minimal clinical difference of 8.0, an alpha of 0.05 and a power of 0.80, resulting in a sample size of 36 patients, 18 in each group. An attrition rate of 25% was added and adjusted for the block size of eight leading to 48 patients needed for final inclusion.

Due to the coronavirus disease 2019 (COVID-19) pandemic, the trial was temporarily closed down for inclusion three times (between 13 March 2020 and 1 September 2020, between 8 December 2020 and 1 May 2021 and between 20 December 2021 and 5 January 2022), on the recommendation of the steering committee consisting of R.D.B., S.O.D., C.J. and I.G. One of the centres (Department of Surgery, Slagelse Hospital) was not able to open for inclusion again after the second close-down. During the third close-down, the sample size and retention were re-evaluated by the principal investigator and steering committee. It was decided to conclude the trial after inclusion of 40 patients, because attrition was lower than expected and this would meet the goal of 36 patients concluding the trial as required in the original sample size calculation.

### Statistics

Continuous data were visually inspected for normal distribution in histograms and QQ-plots. For analysing the primary outcome and other repeated measures a generalized linear mixed model with unstructured covariance was used, and results were presented with 95% c.i. Missing data were assumed to be missing completely at random and handled by maximum likelihood estimation within the linear mixed model. No specific per protocol measure of adherence or compliance was defined and all patients undergoing elective surgery were included in the analysis. In other comparisons between groups a *t*-test was used for continuous variables with acceptable normal distribution and presented with mean(s.d.) and Wilcoxon signed rank test for continuous variables without normal distribution presented with median, i.q.r. and range. Fisher’s exact test and chi-squared test were used for categorical variables and presented with numbers and per cent. The data set did not contain missing data in baseline and treatment characteristics. All statistical analyses were performed in SAS version 9.4 (SAS Institute, North Carolina, USA) and reporting was done according to the CONSORT statement for RCTs^32^.

## Results

**Fig. 1:**
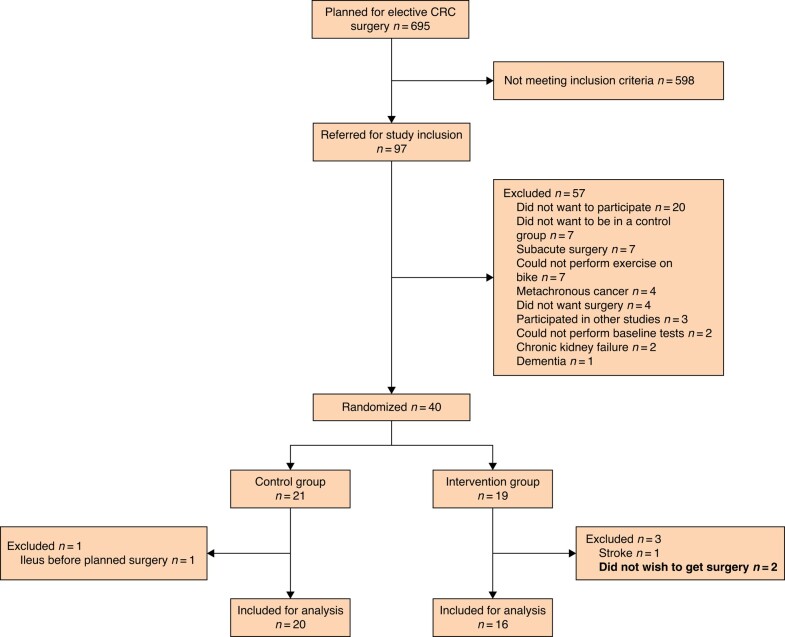
CONSORT diagram describing the inclusion and exclusion process CRC, colorectal cancer

During the study period, a total of 97 patients were referred for inclusion, and of those 40 (41%) were included, baseline tested and randomized (see *Fig. 1* for details and exclusions). Subsequently, four patients were excluded leaving 36 patients (20 in the control group and 16 in the intervention group) for final analysis. Of the four excluded patients after randomization, two did not want to have the surgery performed and withdrew their consent, one was excluded due to a stroke needing hospitalization and subsequent rehabilitation, and one was excluded because of acute-onset large bowel obstruction needing emergency surgery. Differences in patient and treatment characteristics between the groups are presented in *Table 1*. The mean(s.d.) age of included patients was 79(6.5) and the majority of patients had colonic cancers (32 (89%)). Seventeen (47%) male patients were included; however, 11 of these were randomized to the intervention group, leading to an imbalance between the groups regarding sex (*P* = 0.043). No difference between the groups was found in either VO_2_ max or VO_2_ uptake at the anaerobic threshold at baseline (12.6 ml/kg/min (i.q.r. 11.5–14.9) *versus* 12.5 ml/kg/min (i.q.r. 9.4–14.2), and 9.1 ml/kg/min (i.q.r. 7.6–10.7) *versus* 8.3 (i.q.r. 7.1–10.5), respectively). Right-sided hemicolectomies (including extended hemicolectomies) were the predominant operation in both groups with 11 (69%) in the intervention group and 16 (80%) in the control group. The median time between inclusion and surgery was 32 days (i.q.r. 29–34 days) for the intervention group and 24 days (i.q.r. 18–28 days) for the control group (*Table 2*).

**Table 1:** Patient and treatment characteristics of included patients

	Intervention *n* = 16	Control *n* = 20
**Inclusion site**		
ZUH	12 (75)	16 (80)
SLA	4 (25)	4 (20)
**Sex**		
Male	11 (69)	6 (30)
Female	5 (31)	14 (70)
Age (years), mean(s.d.)	80(6.9)	78(6.3)
**ASA score**		
2	4 (25)	6 (30)
3	12 (75)	14 (70)
**WHO performance status**		
1	10 (63)	9 (45)
2	6 (37)	11 (55)
**Cancer**		
Colonic	14 (88)	18 (90)
Rectal	2 (12)	2 (10)
**UICC stage**		
1	10 (63)	7 (35)
2	3 (19)	9 (45)
3	3 (19)	4 (20)
**Smoking status**		
Current smoker	1 (6)	2 (10)
Previous smoker	10 (63)	15 (75)
Non-smoker	5 (31)	3 (15)
Pack-years, median (i.q.r., (range))	19 (0–30 (0–60))	30 (10–45 (0–61))
Alcohol U/W, median (i.q.r., (range))	2 (0–7 (0–14))	0 (0–1 (0–15))
**Co-morbidities**		
Charlson Co-morbidity Index, median (i.q.r., (range))	5 (4–6 (3–7))	5 (4–6 (3–8))
Diabetes	7 (44)	6 (30)
Cardiovascular disease	8 (50)	10 (50)
COPD	3 (19)	8 (40)
Previous abdominal surgery	8 (50)	9 (45)
Polypharmacy (≥5 drugs)	12 (75)	11 (55)
BMI (kg/m^2^), median (i.q.r., (range))	27.9 (24.8–31.4 (18.3–34.5))	26.8 (22.8–32.7 (19–39.8))
Body weight (kg), mean(s.d.)	79.8(15.3)	77.9(19.1)
Weight loss of total body-weight, median (i.q.r., (range))	3 (0–9 (0–13))	3 (0–9 (0–18))
G8 score, median (i.q.r., (range))	12 (10–14 (6–15))	12 (10–13 (6–15))
**Fraility**		
Non-frail	1 (6)	4 (20)
Prefrail (1–2)	9 (56)	7 (35)
Frail 3+	6 (38)	9 (45)

Values are *n* (%) for categorical data unless otherwise stated. Mean(s.d.) for continuous data with normal distribution; median (interquartile range (i.q.r.), (range)) for data without acceptable normal distribution. COPD, chronic obstructive pulmonary disease; SLA, Department of Surgery, Slagelse Hospital; U/W, unit per week; ZUH, Department of Surgery, Zealand University Hospital; UICC, Union for International Cancer Control.

**Table 2:** Intervention characteristics, surgical procedure and postoperative course including enhanced recovery after surgery compliance measures

	Intervention *n* = 16	Control *n* = 20	*P*
**Intervention characteristics**			
Prehab. time (days), median (i.q.r., (range))	32 (29–34 (18–40))	24 (18–28 (7–47))	0.001
No. of patients receiving intravenous iron	12 (75)	15 (75)	1.000
Dosage of intravenous iron (mg), median (i.q.r., (range))	1500 (500–1500 (0–2000))	1350 (500–1800 (0–2000))	0.795
Haemoglobin (mmol/l), mean(s.d.)	6.76(1.08)	6.42(0.93)	0.517
Change in haemoglobin (mmol/l), median (95% c.i.)	0.56 (0.081–1.04)	0.47 (0.036–0.91)	
6-MWT (m), median (i.q.r., (range))	304 (246–395 (133–525))	260 (233–331 (80–441))	0.293
Change in 6-MWT (m), median (95% c.i.)	−7.9 (−40.3–24.5)	37 (7.7–66.5)	
STS 30 s (repetitions), median (i.q.r., (range))	9 (8–12 (1–16))	9 (7–11 (0–13))	0.311
Change in STS 30 s (repetitions), median (95% c.i.)	1.90 (0.80–3.02)	1.61 (0.64–2.59)	
**Surgical procedure**			
Minimally invasive	15 (94)	19 (95)	1.000
Converted to open surgery	1 (6)	1 (5)	
Minutes of surgery, mean(s.d.)	147(46)	161(58)	0.372
Stoma	0	3 (15)	0.238
**Postoperative outcomes**			
LOS (days), median (i.q.r., (range))	4 (3–7 (1–30))	4 (3–7 (2–18))	0.975
DAOH-30 (days), median (i.q.r., (range))	25 (17–27 (0–29))	26 (23–27 (10–28))	0.557
Intensive care	1 (6)	2 (10)	1.000
Reoperation	4 (25)	4 (20)	1.000
Readmission	3 (19)	1 (5)	0.303
Comprehensive complication index, median (i.q.r., (range))	14.4 (0–26.9 (0–100))	14.8 (0–23.6 (0–49.5))	0.681
Complications (Clavien–Dindo*)			0.606
0	8 (50)	7 (35)	
1	1 (6)	3 (15)	
2	2 (13)	6 (30)	
3a	2 (13)	1 (5)	
3b	2 (13)	2 (10)	
4a	0	1 (5)	
4b	0	0	
5	1 (6)	0	
No. of patients with a complication Clavien-Dindo ≥3a	5 (31)	4 (20)	0.470
**ERAS compliance measures**			
Urinary catheter removal POD 0	67	82	0.424
Tolerating oral nutrition POD 1	73	47	0.171
Postoperative day of full mobilization, median (i.q.r., (range))	2 (0–3 (0–6))	2 (1–3.5 (0–14))	0.871

Values are *n* (%) for categorical data unless otherwise stated. Mean(s.d.) for continuous data with normal distribution; median (interquartile range (i.q.r.), (range)) for data without acceptable normal distribution. Changes between baseline testing and preoperative testing provided with 95% c.i. 6-MWT, 6-minutes' walk test; DAOH-30, days alive and out of hospital 30-days; LOS, length of stay; POD, postoperative day; ERAS, enhanced recovery after surgery.

*Highest Clavien–Dindo for each patient.

### Primary outcome

**Fig. 2:**
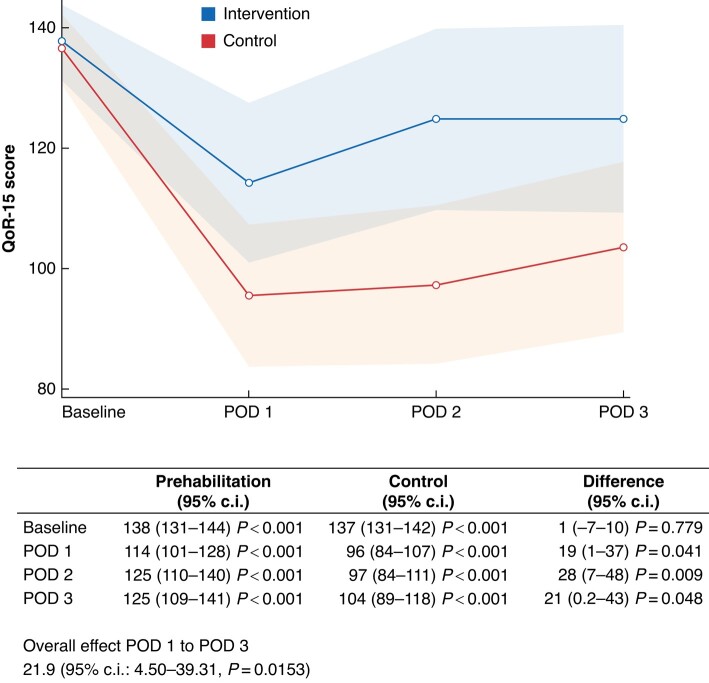
**Results of analysis of the primary outcome quality of recovery 15 (QoR-15) within the first 3 postoperative days (POD)** *X*-axis: QoR-15 score. *Y*-axis: postoperative day. Blue line: intervention group. Red line: control group. Transparent blue and red area: 95% c.i. The table below presents the values and the results of a generalized linear mixed model with unstructured covariance, including the overall effect and differences at each postoperative day between the intervention group and the control group

The primary outcome was the difference in recovery measured by the QoR-15 score during the first 3 postoperative days (*Fig. 2*). In the analysis of the overall effect of prehabilitation using the generalized linear mixed model, the intervention improved the QoR-15 score by 21.9 (95% c.i. 4.50–39.31), during the first 3 postoperative days when compared with the control group. Baseline measurements of QoR-15 were similar in the groups (138 and 137 for the intervention and control groups, respectively). At postoperative day 1 the mean QoR-15 score for the intervention group was 114 (95% c.i. 101–128) compared with 96 (95% c.i. 84–107) for the control group. Similar findings were found for postoperative days 2 and 3, with a mean QoR-15 score of 125 (95% c.i. 110–140) and 125 (95% c.i. 109–141) for the intervention group, and 97 (95% c.i. 84–111) and 104 (95% c.i. 89–118) for the control group, respectively. In the generalized linear mixed model, the intervention was associated with a statistically significant greater improvement in QoR-15 score of 19 (95% c.i. 1–37) at postoperative day 1, 28 (95% c.i. 7–48) at postoperative day 2 and 21 (95% c.i. 0.2–43) at postoperative day 3, compared with the control group.

### Secondary outcomes

Both groups increased their haemoglobin levels and their sit-to-stand test score slightly between the baseline and the preoperative testing. Only the control group improved their 6-MWT by 37 meters (95% c.i. 8–67 m), and a small improvement of −2.4 of the PG-SGA score was found for the intervention group (95% c.i. −3.6 to −1.2). Neither of the groups showed any other statistically significant changes in other measures of physical function, nutritional or blood work (*Table 3*). No statistically significant differences in the postoperative outcome measures were found between the groups. A total of 29 complications were observed (10 in the intervention group and 19 in the control group), however, with no significant difference in number of patients without complications, severity measured by the Clavien–Dindo classification or the comprehensive complication index.

**Table 3:** Additional intervention-specific variables and surgical procedures

	Intervention *n* = 16	Control *n* = 20	*P*
**Physical fitness and function**			
VO_2_ max (ml/kg/min), median (i.q.r., (range))	12.6 (11.5–14.9 (10.1–20.6))	12.5 (9.4–14.2 (5–17.4))	0.228
Change in VO_2_ max (ml/kg/min), median (95% c.i.)	0.63 (−0.46–1.72)	0.67 (−0.37–1.71)	
VO_2_ at AT (ml/kg/min), median (i.q.r., (range))	9.1 (7.6–10.7 (6.7–12.6))	8.3 (7.1–10.5 (1.8–12.4))	0.308
Change in VO_2_ at AT (ml/kg/min), median (95% c.i.)	0.22 (−0.78–1.26)	0.34 (−0.64–1.33)	
Hand grip strength (kg), median (i.q.r., (range))	29.7 (18.5–34.3 (10.6–39.6))	23.1 (18.5–29.4 (14.4–36.1))	0.132
Change in hand grip strength (kg), median (95% c.i.)	0.6 (−0.6–1.8)	1.1 (−0.004–2.2)	
**Nutritional status**			
Change in total body weight (kg), median (95% c.i.)	1.1 (−0.44–2.67)	0.29 (−1.08–1.64)	
PG-SGA score, median (i.q.r., (range))	6.5 (4–8 (2–11))	6.5 (3.5–10 (2–12))	0.663
Change in PG-SGA score, median (95% c.i.)	−2.4 (−3.6 to −1.2)	−1.0 (−2.1–0.04)	
**Bloodwork**			
NLR, median (i.q.r., (range))	4.00 (2.43–4.83 (1.77–36.50))	4.69 (3.56–5.82 (1.95–7.20))	0.257
Change in NLR, median (95% c.i.)	−1.53 (−3.23–0.18)	−0.39 (−1.88–1.11)	
CRP (mg/l), median (i.q.r., (range))	6.65 (3.75–14 (2.9–40))	9.85 (3.6–20.5 (2.9–140))	0.517
Change in CRP (mg/l), median (95% c.i.)	−1.5 (−6.2–3.2)	−0.6 (−4.5–3.26)	
Creatinine (µmol/l), mean(s.d.)	92(30)	81(21)	0.142
Change in creatinine (µmol/l), median (95% c.i.)	−0.13 (−6.1–5.8)	0.07 (−5.4–5.6)	
Albumin (g/l), mean(s.d.)	33(5.0)	32(5.5)	0.674
Change in albumin (g/l), median (95% c.i.)	0.38 (−1.7–2.5)	−0.23 (−2.1–1.7)	
**Type of surgery**			
Laparoscopic right hemicolectomy	8 (50)	13 (65)	
Laparoscopic extended right hemicolectomy	2 (13)	2 (10)	
Open right hemicolectomy	1 (6)	1 (5)	
Laparoscopic left hemicolectomy	2 (13)	1 (5)	
Laparoscopic sigmoid resection	1 (6)	1 (5)	
Laparoscopic rectal resection	2 (13)	2 (10)	

Values are *n* (%) for categorical data unless otherwise stated. Mean(s.d.) for continuous data with normal distribution; median (interquartile range (i.q.r.), (range)) for data without acceptable normal distribution. Changes between baseline testing and preoperative testing provided with 95% c.i. AT, anaerobic threshold; CRP, C-reactive protein; NLR, neutrophil to lymphocyte ratio; PG-SGA, patient-generated subjective global assessment; VO_2_, volume oxygen.

### Other variables

The median adherence and compliance with the training intervention were both 100% (range: 80–100% and 64–100%, respectively). The median compliance with the nutritional supplements was 98% (range: 0–100%). All three patients that smoked at inclusion succeeded in smoking cessation (two in the control group and one in the intervention group). Intravenous iron was administered in both equal frequencies (75%) and dosages between the groups (1500 mg *versus* 1350 mg). Minimally invasive surgery was conducted in 34 of 36 patients (94%) and showed no statistically significant differences in ERAS characteristics within the first 3 postoperative days.

### Adverse events and breach of protocol

Six patients, three in the intervention group and three in the control group, developed a serious adverse event during the preoperative course. Two of these led to discontinuation of participation and one to a breach of protocol. None of these were deemed to be a result of the prehabilitation (see Supplementary material S1). Of the 36 patients concluding the trial, only 17 (47%) patients were able to perform the planned tests 4 weeks after surgery. The most common reasons for not being able to perform the test were postoperative complications (*n* = 9, 25%) and exhaustion due to concomitant adjuvant chemotherapy (*n* = 2, 6%). Only one patient did not show up for the planned testing session. Due to the COVID-19 pandemic and subsequent shut-downs, seven (19%) patients had their postoperative testing sessions cancelled.

## Discussion

The present study aimed to investigate the effect of multimodal prehabilitation on immediate postoperative recovery in patients with low functional capacity. Patients in the intervention group had a 21.9 (95% c.i. 4.5–39.3) point higher recovery score during the first 3 postoperative days when compared with patients in the control group. This difference is above the minimal clinically relevant difference of 8, suggesting that undergoing multimodal prehabilitation can improve immediate postoperative recovery in vulnerable patients with colorectal cancer.

To the authors' knowledge, no previous studies have estimated the effect of prehabilitation in the early postoperative period in any population, which makes interpretation of the results into the clinical setting difficult. However, the improvement found in QoR-15 score associated with the multimodal intervention was of the same magnitude between moderate to good recovery, a difference which in previous studies has shown to reduce the risk of complications and improve resumption of recreational and occupational activities^28^. Only a few other statistically significant results were found, such as the increase in the sit-to-stand test and haemoglobin for both groups, and 6-MWT for the control group. While the increase in haemoglobin can be explained by the high frequencies of administration of intravenous iron^33^ in both groups (75%), the reason for the increase in 6-MWT for the control group is unclear. However, the study was not powered to show any preoperative or postoperative difference between the groups in these outcomes. Conclusions based on the effects on postoperative complications or improvements in the physical tests should be avoided, and the above-mentioned improvements could be the result of multiple testing.

Previous studies have shown that it is possible to improve functional capacity prior to surgery through physical training and to reduce complications^5,7,34–36^. However, this was not the aim of the present study. It is important to note that all patients that presented with iron-deficiency anaemia were treated with intravenous iron in accordance with both local and Danish guidelines for treating colorectal cancer^37^. Treatment with intravenous iron can improve physical fitness^38^ and is an intervention seldom included as part of multimodal prehabilitation. It is interesting that the same improvements were not observed in VO_2_ max, VO_2_ at the anaerobic threshold and body weight, as demonstrated in the preceding feasibility trial^13^ and by other studies in similar populations. While increasing age, co-morbidities and frailty increase the anabolic resistance to anabolic stimuli such as training, it has been proposed that using a multimodal approach could increase functional capacity and physical fitness^2^. A previous RCT by Carli *et al*.^39^ including only frail patients with colorectal cancer (Frailty Score ≥2) did not demonstrate any improvements in 6-MWT prior to surgery. Similar results were found by a recent trial including 204 patients undergoing home-based training prior to cancer surgery^40^. This indicates that additional research is needed to identify which patients can benefit from prehabilitation and whether other interventions that may work in adjunct with training and dietary interventions should be included. No analyses of the postoperative tests are presented, as patients that could not undergo the physical testing post surgery all had postoperative complications or underwent adjuvant oncological treatment. These missing data would be missing not at random and any attempts to impute or analyse these data would be biased^41^.

This study has several important limitations. First, the study participants and testers were not blinded, which may have influenced the results. All baseline testing was conducted prior to randomization, thereby blinding all baseline test results; however, both testers and patients were aware of the overall study hypothesis, which could have led to response bias. Secondly, there was an imbalance between the groups in regards to gender, with more males in the intervention group. While the influence of sex and WHO performance status on the QoR-15 score is unknown, previous studies have shown that both male sex and increasing WHO performance status provide a higher risk of postoperative complications after colorectal cancer surgery^11^, and one of the fundamental assumptions of prehabilitation is that lower functional capacity will lead to slower recovery. The difference in performance status could potentially lead to confounding and should be kept in mind when interpreting the results. However, the same trend with lower functional capacity in the control group was not found in more objective measures of physical fitness such as the VO_2_ max and VO_2_ anaerobic threshold (AT) measurements. Thirdly, the study was temporarily closed down three times during the COVID-19 pandemic, which reduced both the number of included centres and subsequently the sample size. Despite the lockdown, there was no change in recruitment rate, no refusals due to fear of COVID-19 infection, and the delivery of the intervention remained the same. However, because the situation is unprecedented, possible confounders and biases could have arisen. As an example, during the societal lockdown, all training centres were closed and all non-essential services suspended, thereby potentially leading to reduced physical capacity and nutritional status of the patients included after the lockdowns^42^. The impact of previous or concurrent COVID-19 infection and vaccination status during the trial was not registered. One of the major contributing factors of the death observed within the intervention group was infection with COVID-19 just prior to surgery, which has been previously shown to be a significant risk factor for postoperative morbidity and mortality^43^. Only 36 of 97 eligible patients (37%) were included in the study, a common trend in prehabilitation studies. In previous studies investigating prehabilitation in ‘high’-risk patients, Barberan-Garcia *et al*.^34^ and Berkel *et al*.^35^ included 52% and 43% patients, respectively. Non-participation in surgical RCTs despite fulfilling the inclusion criteria is associated with increased severity of disease stage and higher frequency of co-morbidities^44^. This introduces a volunteer bias, which is difficult to avoid in studies that evaluate interventions that require high patient participation and motivation, such as exercise interventions in older adults^45^, which limits the external validity of the results.

The primary strength of the study is the rigid adherence to both minimally invasive surgery and the ERAS care pathway for all of the included patients, reflected in both the surgical treatment, the low occurrence of complications and short length of stay, despite only including ‘high’-risk patients with low functional capacity. The study demonstrates the effect of prehabilitation as an adjunct to the highest standard of care. Study participants had a very high degree of both adherence and compliance with the intervention, with a lower than expected number of dropouts. However, because all patients that discontinued after inclusion in the intervention group did not receive surgery, we were not able to conduct an intention-to-treat analysis, and only a per-protocol analysis was performed.

Further confirmatory studies with larger sample sizes to avoid imbalance issues are needed, as are studies establishing which patients will benefit from prehabilitation as an adjunct to the current ERAS care pathway for colorectal cancer, and to estimate the effect on both postoperative complications and long-term outcomes.

## Supplementary Material

zrad134_Supplementary_Data

## Data Availability

An anonymized version of the original data set and analytic code will be provided on reasonable request by contacting the corresponding author at radb@regionsjaelland.dk for a period of 5 years after publication.

## Protocol

The full protocol was published in the journal *Pilot and Feasibility Studies* (doi:10.1186/s40814-022-00967-8)
