# Supplementary material for: Preoperative multimodal prehabilitation before elective colorectal cancer surgery in patients with WHO performance status I or II: randomized clinical trial
Source: BJS Open. 2023 Dec 7;7(6):zrad134. doi: 10.1093/bjsopen/zrad134 (PMC10702628; doi:10.1093/bjsopen/zrad134)
Supplement: zrad134_Supplementary_Data [file zrad134_supplementary_data.docx]

**Supplementary material**

**Preoperative Multimodal prehabilitation prior to elective colorectal cancer surgery in patients with WHO performance status I or II: Randomized clinical trial**

Authors

*Rasmus Dahlin Bojesen, MD, Ph.d.^1,2^*

*Susanne Oksbjerg Dalton, Professor, Ph.d.^3,4^*

*Søren Thorgaard Skou, Professor, Ph.d.^5,6^*

*Lars Bo Jørgensen, MSc..^5,6,7^*

*Line Rosell Walker, MD^1^*

*Jens Ravn Eriksen, MD, Ph.d.^2,8^*

*Camilla Grube, MD^1,2^*

*Tobias Freyberg Justesen, MD.^2^*

*Christoffer Johansen, Professor, DmSc.^4,9^*

*Gerrit Slooter, MD, Ph.d.^10^*

*Franco Carli, Professor, MPhil, FRCA, FRCPC^11^*

*Ismail Gögenur, Professor, DmSc.^2^*

^1^ Department of Surgery, Slagelse Hospital, Slagelse, Denmark

^2^ Center for Surgical Science, Zealand University Hospital, Køge, Denmark

^3^ Department of Clinical Oncology, Zealand University Hospital, Næstved, Denmark

^4^ The Danish Cancer Society Research Center, Copenhagen, Denmark

^5^ Research Unit for Musculoskeletal Function and Physiotherapy, Department of Sports Science and Clinical Biomechanics, University of Southern Denmark, Odense, Denmark

^6^ The Research Unit PROgrez, Department of Physiotherapy and Occupational Therapy, Naestved-Slagelse-Ringsted Hospitals, Slagelse, Denmark

^7^ Department of Physiotherapy and Occupational Therapy, Zealand University Hospital, Roskilde, Denmark

^8^ Department of Surgery, Zealand University Hospital, Køge, Denmark

^9^ Late Effect Research Unit CASTLE, Finsen Center, Rigshospitalet, Copenhagen, Denmark

^10^ Department of Surgery, Maxima Medical Center, Eindhoven, The Netherlands

^11^ Department of Anesthesia, Faculty of Medicine and Health Sciences, McGill University, Montreal, Canada

**Corresponding author.**

Rasmus Dahlin Bojesen, MD, Ph.d.

Department of Surgery, Zealand University Hospital

Lykkebækvej 1

DK-4600 Køge

[radb@regionsjaelland.dk](mailto:radb@regionsjaelland.dk) and [rasmus.bojesen@gmail.com](mailto:rasmus.bojesen@gmail.com)

Orchid ID: 0000-0003-3998-1160

**Supplementary Materials - Index**

| **Supplementary Methods** |  |
| --- | --- |
| Consort checklist | *pag. 3* |
|  |  |
| **Supplementary Results** |  |
|  | *pag. 4* |
|  |  |
|  |  |
|  |  |
|  |  |
|  |  |
|  |  |
|  |  |
|  |  |
|  |  |

**Supplementary Methods**

| Section/Topic | Item No | Checklist item | Reported on page No |
| --- | --- | --- | --- |
| Title and abstract | | | |
|  | 1a | Identification as a randomised trial in the title | 1 |
|  | 1b | Structured summary of trial design, methods, results, and conclusions (for specific guidance see CONSORT for abstracts) | 3 |
| Introduction | | | |
| Background and objectives | 2a | Scientific background and explanation of rationale | 4 |
|  | 2b | Specific objectives or hypotheses | 4 |
| Methods | | | |
| Trial design | 3a | Description of trial design (such as parallel, factorial) including allocation ratio | 4-5 |
|  | 3b | Important changes to methods after trial commencement (such as eligibility criteria), with reasons | 7 |
| Participants | 4a | Eligibility criteria for participants | 5 |
|  | 4b | Settings and locations where the data were collected | 4 |
| Interventions | 5 | The interventions for each group with sufficient details to allow replication, including how and when they were actually administered | 5+trial paper |
| Outcomes | 6a | Completely defined pre-specified primary and secondary outcome measures, including how and when they were assessed | 6 |
|  | 6b | Any changes to trial outcomes after the trial commenced, with reasons | NA |
| Sample size | 7a | How sample size was determined | 7 |
|  | 7b | When applicable, explanation of any interim analyses and stopping guidelines | 7 |
| Randomisation: |  |  |  |
| Sequence generation | 8a | Method used to generate the random allocation sequence | 6 |
|  | 8b | Type of randomisation; details of any restriction (such as blocking and block size) | 6 |
| Allocation concealment mechanism | 9 | Mechanism used to implement the random allocation sequence (such as sequentially numbered containers), describing any steps taken to conceal the sequence until interventions were assigned | 6 |
| Implementation | 10 | Who generated the random allocation sequence, who enrolled participants, and who assigned participants to interventions | 6 |
| Blinding | 11a | If done, who was blinded after assignment to interventions (for example, participants, care providers, those assessing outcomes) and how | 6 |
|  | 11b | If relevant, description of the similarity of interventions | NA |
| Statistical methods | 12a | Statistical methods used to compare groups for primary and secondary outcomes | 7 |
|  | 12b | Methods for additional analyses, such as subgroup analyses and adjusted analyses | NA |
| Results | | | |
| Participant flow (a diagram is strongly recommended) | 13a | For each group, the numbers of participants who were randomly assigned, received intended treatment, and were analysed for the primary outcome | 8 + figure 2 |
|  | 13b | For each group, losses and exclusions after randomisation, together with reasons | 8 + figure 2 |
| Recruitment | 14a | Dates defining the periods of recruitment and follow-up | 4+8 |
|  | 14b | Why the trial ended or was stopped | 7 |
| Baseline data | 15 | A table showing baseline demographic and clinical characteristics for each group | Table 1 |
| Numbers analysed | 16 | For each group, number of participants (denominator) included in each analysis and whether the analysis was by original assigned groups | 8-9+ table 1+ table 2 + figure 3 |
| Outcomes and estimation | 17a | For each primary and secondary outcome, results for each group, and the estimated effect size and its precision (such as 95% confidence interval) | 8-9 + figure 3 |
|  | 17b | For binary outcomes, presentation of both absolute and relative effect sizes is recommended | NA |
| Ancillary analyses | 18 | Results of any other analyses performed, including subgroup analyses and adjusted analyses, distinguishing pre-specified from exploratory | NA |
| Harms | 19 | All important harms or unintended effects in each group (for specific guidance see CONSORT for harms) | 9 |
| Discussion | | | |
| Limitations | 20 | Trial limitations, addressing sources of potential bias, imprecision, and, if relevant, multiplicity of analyses | 10-11 |
| Generalisability | 21 | Generalisability (external validity, applicability) of the trial findings | 9-11 |
| Interpretation | 22 | Interpretation consistent with results, balancing benefits and harms, and considering other relevant evidence | 9-11 |
| Other information | | |  |
| Registration | 23 | Registration number and name of trial registry | 2+11 |
| Protocol | 24 | Where the full trial protocol can be accessed, if available | 11 |
| Funding | 25 | Sources of funding and other support (such as supply of drugs), role of funders | 2+11 |

**Supplementary Figures and Tables**

**Supplementary table 1 (S1):** Individual description of serious adverse events.

| Group | Adverse event | Description | Consequence |
| --- | --- | --- | --- |
| Intervention | Urinary tract infection | Participant known to have repetitive urinary tract infections. Developed usual symptoms without fever. | Treated with antibiotics and could continue with the intervention |
| Intervention | Stroke | Developed apoplexy due to a blood clot, outside training sessions | Discontinuation of the intervention. Excluded from analysis |
| Intervention | Tooth abscess, endocarditis and COVID infection | After completion of the training intervention but before surgery developed a tooth abscess and subsequent endocarditis needing hospitalization. Contracted COVID-19 during the admission and surgery was total postponed 4 weeks. | Breach of protocol. Included in the analysis, despite the prolonged time between prehabilitation and surgery. Died postoperatively due to respiratory failure and possible aspiration. |
| Control | Hip fracture | Fell and broke hip | Had emergency orthopaedic surgery, and primary surgery postponed until fit (4 weeks). |
| Control | Ileus | Developed stenosis of the tumour awaiting surgery. A subacute colectomy was performed. | Excluded from analysis |
| Control | Esophagitis | Presented with swallowing difficulties, shortly after inclusion. | A gastroscopy was performed and treated with fluconazole and pantoprazole. |

**References**
